# Human Mesenchymal Stem Cells Provide Protection against Radiation-Induced Liver Injury by Antioxidative Process, Vasculature Protection, Hepatocyte Differentiation, and Trophic Effects

**DOI:** 10.1155/2013/151679

**Published:** 2013-11-28

**Authors:** Sabine Francois, Moubarak Mouiseddine, Bénédicte Allenet-Lepage, Jan Voswinkel, Luc Douay, Marc Benderitter, Alain Chapel

**Affiliations:** ^1^Laboratoire de Radiopathologie et de Thérapies Expérimentales (LRTE), Institut de Radioprotection et de Sûreté Nucléaire (IRSN), PRP-HOM/SRBE/LRTE, BP 17, F-92262 Fontenay-aux-Roses Cedex, France; ^2^UMRS-938, Equipe Prolifération et Différenciation des Cellules Souches du Pr Luc Douay: Application à la Thérapie Cellulaire, Faculté de Médecine Saint-Antoine, Université Paris VI Pierre et Marie Curie, 27 rue de Chaligny, 75012 Paris, France; ^3^Service d'Hématologie et de Thérapie Cellulaire, Hôpital Saint-Antoine, 75012 Paris, France

## Abstract

To evaluate the potential therapeutic effect of the infusion of hMSCs for the correction of liver injuries, we performed total body radiation exposure of NOD/SCID mice. After irradiation, mir-27b level decreases in liver, increasing the directional migration of hMSCs by upregulating SDF1**α**. A significant increase in plasmatic transaminases levels, apoptosis process in the liver vascular system, and in oxidative stress were observed. hMSC injection induced a decrease in transaminases levels and oxidative stress, a disappearance of apoptotic cells, and an increase in *Nrf2*, *SOD* gene expression, which might reduce ROS production in the injured liver. Engrafted hMSCs expressed cytokeratin *CK18* and *CK19* and *AFP* genes indicating possible hepatocyte differentiation. The presence of hMSCs expressing *VEGF* and *Ang-1* in the perivascular region, associated with an increased expression of *VEGFr1, r2* in the liver, can confer a role of secreting cells to hMSCs in order to maintain the endothelial function. To explain the benefits to the liver of hMSC engraftment, we find that hMSCs secreted *NGF*, *HGF,* and anti-inflammatory molecules *IL-10*, *IL1-RA* contributing to prevention of apoptosis, increasing cell proliferation in the liver which might correct liver dysfunction. MSCs are potent candidates to repair and protect healthy tissues against radiation damages.

## 1. Introduction

Multipotent stromal cells, also named mesenchymal stromal cells or mesenchymal stem cells (MSCs), are capable of dividing and their progenies are further capable of differentiating into one of several mesenchymal phenotypes, such as osteoblasts, chondrocytes, myocytes, marrow stromal cells, tendon-ligament fibroblasts, and adipocytes [1]. Animal models have shown that MSCs can engraft and distribute to several tissues after systemic infusion [2–7] and engraft in several injured tissues [3, 4, 8–13], for example, the liver [14–16]. Previously, we showed that in a mice model the presence of intravenously injected MSCs increased in damaged tissues following radiation exposure [7, 8] and that in a nonhuman primate model MSCs could be detected in regenerating tissues [4]. Thanks to their relatively easy isolation from bone marrow (BM) and to their extensive capacity for in vitro expansion, MSCs have been considered for approaches in cell therapy and tissue engineering [17–19]. A number of clinical trials are ongoing to explore the effect of MSCs in vivo in several contexts, such as facilitation of hematopoietic recovery after hematopoietic stem cell transplantation (HSCT) [5, 20–22], prevention and treatment of graft-versus-host disease (GVHD) [23, 24], and treatment of osteogenesis imperfecta [25, 26] and metabolic disorders [27]. It has been shown that MSCs infusion engraftment in the liver facilitates recovery from chemically induced acute liver damage as well as recovery by an indirect effect after radiation injury [28–32]. In addition, these MSCs differentiate in hepatocyte-like cells and secrete a variety of cytokines and growth factors that have both paracrine and autocrine activities. These secreted bioactive factors suppress the local immune system, inhibit fibrosis (scar formation) and apoptosis, enhance angiogenesis, and stimulate mitosis and differentiation of tissue-intrinsic reparative cells and stem cells [33]. MSCs are promising candidates for the repair of tissues altered by radiation exposure, as described for skin regeneration [8, 9]. Additionally, MSCs have antiproliferative, immune-modulatory, antioxidative, and anti-inflammatory effects [28–34]. MSCs have implications for treatment of allograft rejection, graft-versus-host disease, autoimmune inflammatory bowel disease, and other disorders in which immunomodulation and tissue repair are required.

Bone marrow transplantation (BMT) is a sophisticated therapeutic procedure consisting in high-dose chemo-radiotherapy followed by intravenous infusion of hematopoietic stem cells to reestablish marrow function. BMT is largely used in treatments of hematologic malignancies, including leukemia and lymphomas [35]. This treatment requires conditioning consisting in a massive chemotherapy combined, or not, with total body irradiation (TBI). Before the BMT, the TBI is performed by ionizing radiation (IR) inducing the release of free radicals in tissues [36]. Thus, IR can damage both the healthy tissues and the tumoral cells which may induce secondary effects due to radiation exposure [37].

Liver disease is an important cause of morbidity among BMT recipients. A retrospective study realized on a group of 103 transplanted patients revealed that the incidence of liver failure attributed to hepatic GVHD was 22.3% and to venoocclusive disease (VOD) was 9.7% [38]. VOD in the liver is a major complication of BMT [39, 40]. GVHD of the liver after allogeneic hematopoietic stem cell transplantation classically presents with increased bilirubin and alkaline phosphatase (ALP) levels. A hepatitic variant presenting more than a 10-fold increase in aspartate aminotransferase (AST) and alanine aminotransferase (ALT) levels was recently recognized [41]. Finally, human mesenchymal stem cell transfusion has demonstrated to improve liver function in acute-on-chronic liver failure patients [42].

The purpose of this study was to reduce the liver toxicity associated with a traditional preparative regimen consisting in massive chemotherapy treatment combined with TBI before BMT. We used an immunotolerant mice model (NOD/SCID mice) receiving a sublethal dose (3.2 Gy) of TBI to observe the biological effect of hMSCs on the induced hepatitic dysfunction. We established a protective role of hMSCs on the liver by limiting the decrease in hepatic activity and the oxidative stress induced by TBI. HMSCs were preferentially localized around the blood vessels of the liver suggesting that hMSCs could ensure the protection of the vascular endothelium against toxic damage. The protection of organ vascular endothelium integrity against free radicals damage by means of MSC infusion could be a potential therapeutic treatment for preventing radiation-induced vascular complications. Therefore, MSC therapy should be considered early on to prevent the progression of liver disease induced by radiation exposure.

## 2. Material and Methods

### 2.1. Isolation, Purification, and Expansion of Human Bone Marrow-Derived MSC (hMSC)

BM cells were obtained from iliac crest aspirates from ten healthy volunteers after informed consent was obtained and used in accordance with the procedures approved by the human experimentation and ethics committees of Saint Antoine Hospital. (The local ethics committee is named “Comité de Protection des Personnes- Ile-de-France V,” Hôpital Saint-Antoine, Paris.) The ethics committee approved this study specifically. Oral patient agreements were obtained before study. This consent procedure was approved by the local committee. The ethics committee did not require the investigator to obtain signed informed consent from all participants because it considered there was no breach of confidentiality, minimal risk for participants, and no procedures involved for which written consent is normally required outside the research context.

As previously described [7], 50 mL of BM was taken from the donors over heparin (Choay by Sanofi-Synthélabo). Low-density mononuclear cells (MNCs) were separated on Ficoll Hypaque density gradient (*d*: 1.077). MNCs were plated at a concentration of 10^7^ cells per milliliter in T-75 cm^2^ tissue culture flasks in Dexter medium (McCoy 5A medium supplemented with 12.5% heat-inactivated fetal calf serum, 12.5% heat-inactivated horse serum, 1% sodium bicarbonate, 1% sodium pyruvate, 0.4% minimum essential medium (MEM) nonessential amino acids, 0.8% MEM essential amino acids, 1% MEM vitamin solution, 1% L-glutamine (200 mM), 1% penicillin-streptomycin solution (all from Invitrogen, Groningen, The Netherlands), 10^6^ M hydrocortisone (Stem Cell Technologies, Vancouver, BC), and 2 ng/mL human basic recombinant fibroblast growth factor (FGFb; R & D Systems, Abington, United Kingdom) and incubated at 37°C and 5% CO_2_ in a humidified atmosphere. After 1 week, nonadherent cells were removed with (the same) complete fresh medium (without hydrocortisone) and first passage hMSCs (P1 hMSC) were plated at a density of 4.10^5^ per T-75 cm^2^ flask. First pass hMSCs (P1 hMSC) were collected and counted when 80% of the cells were confluent. The viability was assessed by trypan blue assay.

After the second passage, hMSCs were plated at different densities using different culture conditions in order to induce osteogenic, chondrogenic, or adipogenic differentiation. Osteogenic differentiation of hMSCs was as follows: cells were plated at a density of 3.10^3^ cells/cm^2^ in Dulbecco's modification of Eagle's medium (DMEM) with L-glutamine supplemented with 10% of FBS (Life Technologies), containing 50 *μ*g/mL of ascorbate-2-phosphate (Sigma) and 100 *μ*M of *β*-glycerolphosphate (Sigma Chemicals Co., Saint Louis, MO, USA). Chondrogenic differentiation of hMSCs was as follows: cells were plated at 50.10^3^ cell/cm^2^ in DMEM with L-glutamine supplemented with 10% of FBS, containing 50 *μ*g/mL of ascorbate-2-phosphate (Sigma) and 10 ng/mL of transforming growth factor TGF-*β*1 (Sigma). Adipogenic differentiation of hMSCs was as follows: cells were plated at 20.10^3^ cells/cm^2^ in DMEM with L-glutamine supplemented with 10% of FBS until confluence. After confluence, the basic media were completed by 50 *μ*g/mL of ascorbate-2-phosphate (Sigma). During culture differentiation, cells were incubated at 37°C with 5% of CO_2_ for 21 days. The medium was renewed every 3 days.

Prior to transplant, a sample of the prepared hMSCs was analyzed by a fluorescence-activated cell sorter (FACS). Following trypsin treatment, human cells were washed and resuspended in aliquots of 2 × 10^5^ cells in phosphate buffered saline (PBS) solution supplemented with 0.5% bovine serum albumin (BSA; Sigma). Staining was performed with phycoerythrin (PE)-conjugated monoclonal antibody against CD105 (SH2), CD73 (SH3), and CD45 (Becton-Dickinson) for 30 minutes at 4°C followed by 2 washes in PBS containing 0.5% BSA. Cells were resuspended in PBS 0.5% BSA and analyzed at 10,000 events/test by FACScalibur (BD Pharmingen). Mouse Immunoglobulin G1 (IgG1) was used as isotopic controls (IOTest). Before infusion when the hMSCs were collected at the second passage, the rates of viability to blue trypan were 98%.

### 2.2. BM-MSC Infusion into NOD/SCID Mouse Model

All experiments and procedures were carried out in accordance with the Guide for the Care and Use of Laboratory Animals as published by the US National Institutes of Health (NIH Publications number 85-23, revised 1996) and with the European Directive number 86/609/CEE and were approved by the local ethics committee (P09-11). NOD-LtSz-*scid/scid* (NOD-SCID) mice, from breeding pairs originally purchased from Jackson Laboratory (Bar Harbor, ME, USA), were bred in our pathogen-free unit and maintained in sterile microisolator cages. TBI was carried out with a sublethal dose of 3.2 Gy (IBL 637, IRSN France) using a source of Cesium (^137^Cs) with a dose rate of 1.85 Gy/minute. We initially investigated the deterioration of hepatic function induced by TBI to determine whether injection of hMSCs could decrease the damages. A total of 100 eight-week-old mice were used to study blood parameters, divided in 4 groups: 25 animals were used as controls (no irradiation, no injection—Group 1), 25 mice were injected with hMSCs but were not irradiated (Group 2), 25 mice received TBI of 3.2 Gy (Group 3), and 25 mice were irradiated (TBI at 3.2 Gy) and injected with hMSCs (Group 4). The mice were put down to collect the blood and the liver at different times (3, 7, 15, 30, and 60 days after TBI). For each of the five time points, 5 animals were used. The BM-MSCs were delivered to each mouse intravenously (IV) through the tail vein using a Myjector 1 mL syringe (TERUMO 29G X 1/2) 24 hours after TBI. The NOD/SCID mice were transplanted with a dose of 5.10^6^ P2 hMSCs in 100 *μ*L of PBS 1X. On average, the hMSCs derived from one bone marrow sample could be were used to inject 5 mice.

### 2.3. Blood Parameters

The plasmatic levels of urea, creatinine, and transaminase were measured to demonstrate the negative impact of TBI on the kidneys and/or the liver. In our first investigation, we observed that plasmatic urea levels decreased significantly between 7 and 15 days after TBI, suggesting a reduction in the hepatic activity (data not shown). We therefore examined the hepatic activity further. We measured the levels of transaminases, urea, and creatinine specifically at 7 days after TBI in the plasma of the 4 groups of mice. The quantitative determination of the plasmatic concentration of urea, creatinine, and transaminases was carried out by means of a Thermo Clinical Labsystems Analyzer (Konelab 20). The analyses were performed on plasma diluted to a final volume of 400 *μ*L. Plasma was obtained from an intracardiac puncture carried out under anesthesia before the liver was carefully collected. The weight and water and food intake of the animals were monitored during the entire study.

### 2.4. Lipid Peroxidation Detection

Lipid peroxidation was assayed by the measurement of related substances that react with thiobarbituric acid (TBARS) [43]. The V79-4 cells were seeded in a culture dish at a concentration of 1 × 105 cells/mL and treated with esculetin at 10 *μ*g/mL after 16 h plating and then hydrogen peroxide (H_2_O_2_) 1 mmol/L was added to the plate after 1 h, which was incubated for a further 24 h. The cells were then washed with cold PBS, scraped, and homogenized in ice-cold 1.15% potassium chloride (KCl). About 100 *μ*L of cell lysates was combined with 0.2 mL of 8.1% SDS, 1.5 mL of 20% acetic acid (adjusted to pH 3.5), and 1.5 mL of 0.8% thiobarbituric acid (TBA). The mixture was adjusted to a final volume of 4 mL with distilled water and heated to 95°C for 2 h. After cooling to room temperature, 5 mL of a mixture of n-butanol and pyridine (15 : 1) was added to each sample, and the mixture was shaken vigorously. After centrifugation at 1,000 ×g for 10 min, the supernatant fraction was isolated, and the absorbance was measured at 532 nm.

### 2.5. Detection and Quantitative Analysis of Engrafted hMSCs: DNA Extraction and PCR Analysis

Biological samples (liver) were submitted for DNA extraction and PCR analysis to detect the presence of human cells in mice recipients. Genomic DNA for PCR analysis was prepared from tissues using the QIAamp DNA mini kit (Qiagen). Amplifications were performed following the standard recommended amplification conditions (Applied Biosystems, Foster City, CA, USA) as previously described by François and colleagues [7]. The amount of DNA contained in each somatic cell (diploid) was 6.16 pg as determined from a single-copy gene. This value was used to calculate the number of gene copies contained in a defined amount of human or mouse DNA (measured by PCR). Therefore, the DNA amount and gene copy number were proportional to the number of cells. The ratio of human DNA over mouse DNA represents the number of human cells in mouse tissues. Amplification of the human beta-globin gene was used to quantify the amount of human DNA in each mouse tissue sample. The endogenous mouse receptor-associated proteins of the synapse (RAPSYN) encoding gene were also amplified, as an internal control. Standard curves were generated for the human beta-globin and mouse RAPSYN genes and used to quantify the absolute amount of human DNA in each mouse tissue. The specificity of human beta-globin amplification was evaluated using tenfold dilution from 100 ng to 0.05 ng of hMSCs DNA with mouse DNA and did not demonstrate cross-reactivity. One hundred nanograms of purified DNA from various tissues was amplified using TaqMan Universal PCR Master Mix (Applied Biosystems). The primer sequences for human beta-globin amplification were 5′GTGCACCTGACTCCTGAGGAGA3′ (forward) and 5′CCTTGATACCAACCTGCCCAGG3′ (reverse), with the fluorescently labeled probe: 5′FAM-AAGGTGAACGTGGATGAAGTTGGTGG-TAMRA-3′. The primer sequences for mouse RAPSYN amplification were 5′ACCCACCCATCCTGCAAAT3′ (forward) and 5′ACCTGTCCGTGCTGCAGAA3′ (reverse), with the fluorescently labeled probe: 5′VIC-CGGTGCCAGTGATGAGGTTGGTCA-TAMRA3′. In order to determine the efficiency of the amplification and the assay precision, calibration curves for human beta-globin and mouse RAPSYN genes were generated with a 0.99 correlation coefficient and efficiency greater than 98%. Mouse DNA was isolated from the identical tissues of nontransplanted NOD/SCID mice and used as a negative control. Likewise, human DNA was isolated from hMSC culture and used as a positive control. The results were expressed in number of human cells per 100 mouse cells in each tissue (directly related to the number of copies of human beta-globin and mouse RAPSYN genes).

### 2.6. Real-Time Quantitative RT-PCR and Detection of miRNA

After isolation of total RNA from cells with RNA mini kit (Qiagen, Courtaboeuf, France), mRNA integrity was checked by analysis with the Agilent 2100 Bioanalyzer (Agilent Technologies, Massy, France). Complementary DNA was constructed by reverse transcription (RT) with SuperScript (Invitrogen). Polymerase chain reaction (PCR) assays were performed using the SYBR PCR Master Mix or TaqMan PCR Master Mix (Life Technologies) and specific primers for selected genes (see Table 1) on an ABI Prism 7900 Sequence Detection System (Invitrogen). For each sample, the PCR fluorescent signal from each target gene was normalized to the fluorescent signal obtained from the housekeeping gene glyceraldehyde 3-phosphate dehydrogenase (GAPDH). The miRNA expression was normalized with U6.

### 2.7. Immunohistochemistry

After paraformaldehyde fixation, organs were rinsed with distilled water and dehydrated. Blocks were cut at 5 *μ*m on a Rotary Microtome (LEICA). For immunohistochemical staining of the paraffin embedded samples, microtomed sections were deparaffinized in xylene and rehydrated in ethanol baths and PBS. The sections were dipped into PBS-triton in order to increase the tissue permeability and were then rinsed for 5 minutes in a distilled water bath. Negative controls were incubated with rabbit IgG diluted to 1 : 100. Detection of bound primary antibody was performed by incubation with biotinylated secondary antibody for 8 minutes. The biotinylated antirabbit IgG secondary antibody composed was diluted to 1 : 200 in PBS1x. Immunohistochemistry (IHC) is a powerful tool allowing detection and visualization of human *β*2-microglobulin within the cell or on the cell surface by traditional light microscopy. The immunoreactivity was performed on a NEXES IHC automat (Ventana, Illkirch, France) using alkaline phosphatase reaction with a FARED substrate detection kit (Enhanced V-red Detection red, number 760031, Ventana) with a 2% trypsin digestion step for 30 minutes (exposing masked epitopes). Slides were incubated for 30 minutes. The polyclonal anti-*β*-2-microglobulin antibody (product NCL-B2Mp, Novocastra) was added at a dilution of 1 : 50. For antibody detection, the Ventana kit was used, followed by counterstaining with hemalyn for 4 minutes. This procedure was controlled by NEXES 8 software. On successive sections, we carried out a HES staining.

### 2.8. Sex Chromosomes FISH and Costaining

Cells were placed on slides and fixed with methanol and acetic acid (3 : 1). The slides were then denatured with 70% formamide in 2 × SSC buffer at 65°C, dehydrated, and air-dried. The probes for the mouse transcription centromere were denatured at 65°C, applied to the denatured slides, and allowed to hybridize in a humid chamber overnight at 37°C in a dark room. The hybridized slides were then washed, and the cells were permeabilized with 0.2% triton-X 100 and incubated with 10% normal serum. Detection of bound primary antibody (human *β*2-microglobulin) was performed by incubation with biotinylated secondary antibody for 8 minutes. The biotinylated antirabbit IgG secondary antibody composed was diluted to 1 : 200 in PBS1x. For controls, we omitted one or both primary antibodies. 

### 2.9. Statistical Analysis

To determine the effect of infusion of hMSCs on hepatic activity after TBI, the rates of urea, creatinine, and transaminases were compared using Student's *t*-test in SigmaStat software. The significance for all analyses was set at *P* < 0.05. All values were expressed as the mean and standard error of the mean (SEM). Each group was composed of 5 to 10 mice according to the study.

## 3. Results 

### 3.1. Mesenchymal Stem Cells Characterization

Phenotypic analysis showed that the hMSCs used in these experiments were strongly positive for the specific surface antigens CD73 and CD105, respectively, 84% ±1.3 and 61% ±1.2. Almost no contamination (0.2% ±0.02 CD 45+ cells) by hematopoietic cells was evidenced in the samples (Table 2).

**Figure 1:**
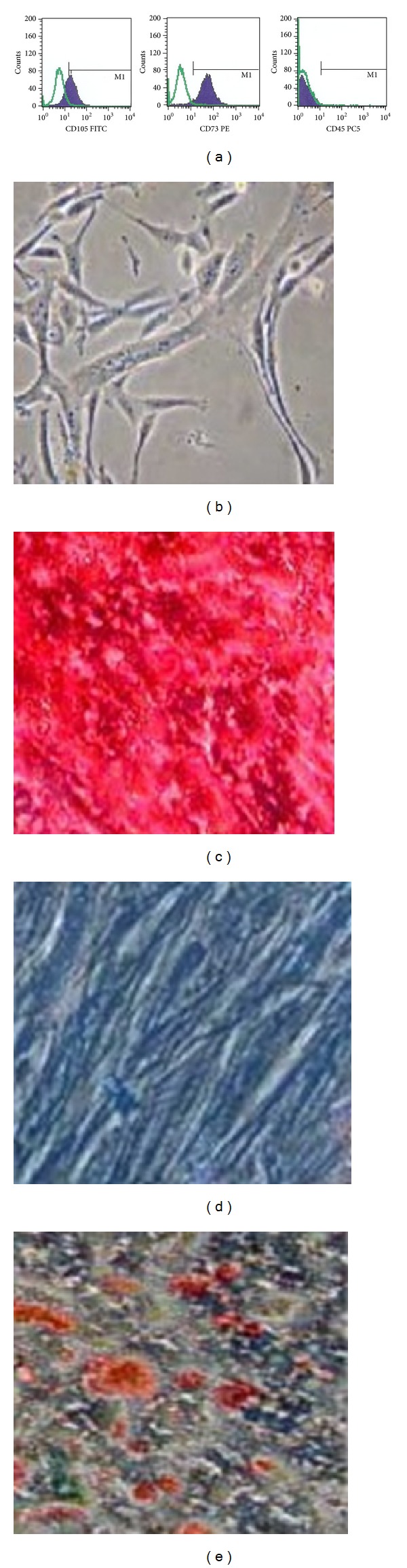
Phenotypic analysis at second passage and in vitro differentiation of hMSCs: FACS analysis of CD105, 73, 45 (a). The cultured hMSCs could enter different cell lineages such as osteogenic (c), chondrogenic (d), and adipogenic (e) lineages. Undifferentiated control (b).

MSCs were morphologically defined by a fibroblast-like appearance. Before use, each batch of MSCs was further characterized by confirming their specific ability to undergo osteogenic, chondrogenic, and adipogenic differentiations (Figure 1). Our results suggest that the hMSCs used for transplant have been expanded without significant loss in their differentiation capacities.

### 3.2. Blood Parameters after TBI

**Figure 2:**
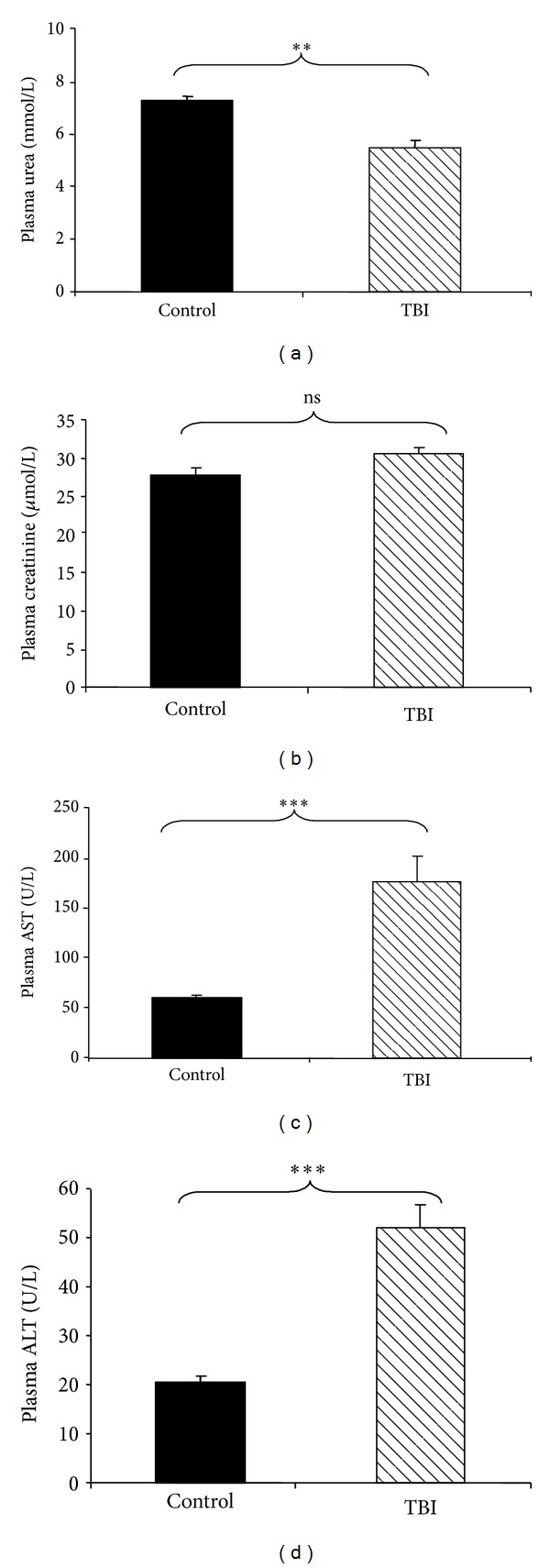
Biochemical analysis of plasma samples at 7 days after TBI. Biochemical analyses of plasmatic urea, creatinine, and transaminases were performed on 200 *μ*L of plasma taken on NOD/SCID mice subjected to TBI at 3.2 Gy. Abbreviations: ALT: serum alanine aminotransferase; AST: serum aspartate aminotransferase. (a) Decrease of plasmatic urea levels at 7 days after TBI. (b) Plasmatic creatinine levels at 7 days after TBI. ((c) and (d)) Increase of plasmatic AST and ALT, respectively, at 7 days after TBI. All values are expressed as the mean and the standard error of the mean (SEM). The significance for all analyses was set at *P* < 0.01 (∗∗) and *P* < 0.001 (∗∗∗), and nonsignificance was noted as ns. Each group was composed of 5 animals (*n* = 5).

The weight and the water and food intake of the animals were monitored for 30 days after TBI. The animals lost weight significantly from 72 hours after radiation exposure. Their weight was normal again three weeks after TBI. No modification of water and food intake was observed for this period. The plasmatic urea level was measured every day following TBI over a period of 30 days. TBI induced a 1.3-fold decrease in blood urea at 7 and 15 days after TBI (*P* < 0.001) (Figure 1(a)). After 15 days after TBI, we noted that blood urea reached its preirradiation level. At 30 days after TBI, the urea level was comparable to that of the controls (7.26 ± 0.18 mmol/L). These observations suggest a decrease in hepatic activity induced by radiation exposure. Thus, we also measured the other blood biochemical parameters, that is, creatinine (Figure 2(b)) and transaminases at 7 days after TBI (Figures 2(c) and 2(d)). The biochemical analysis between irradiated animals and control animals 7 days after TBI showed an increase in blood AST and ALT, while blood creatinine did not vary (30.60 ± 0.87 *μ*mol/L). The absence of an increase in blood creatinine concentration suggests that the TBI had not induced kidney damage 7 days after TBI. However, TBI induced a 2.7-fold increase in blood AST (*P* < 0.001) and a 2.5-fold increase in blood ALT (*P* < 0.001). These observations reveal a negative effect of radiation exposure on the metabolism of the animals. In nonirradiated NOD/SCID mice, the AST/ALT ratio was 3.0 ± 0.2. Seven days after TBI, the AST/ALT ratio was 3.3 ± 0.3. Both transaminases increased at the same time; no transaminase level increased alone. The comparison of the AST/ALT ratio between controls and irradiated animals showed that this ratio did not vary, suggesting that TBI involved hepatic and not muscular injuries. Previously, we reported that 15 days after TBI at 3.5 Gy TBI, cellular depletion of the spleen and hemorrhage in the bone marrow were observed, while no injury was observed in nonirradiated tissues [7]. Comparative histopathological analyses between nonirradiated and irradiated mouse livers at different times after TBI showed no morphological difference. Thus, no injury of hepatic tissue was observed from 3 to 30 days after TBI at 3.2 Gy (data not shown).

### 3.3. Blood Parameters after TBI and BM-MSCs Infusion

**Figure 3:**
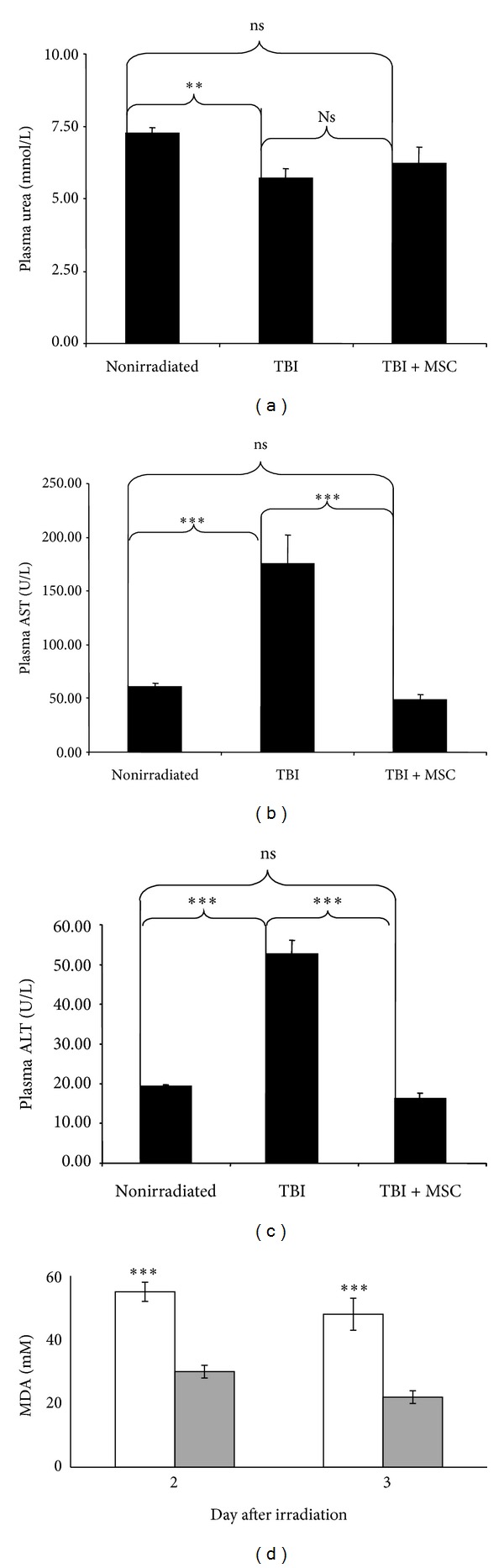
Biochemical analyses of plasmatic urea, creatinine, transaminases, and MDA. 200 *μ*L of plasma was collected from NOD/SCID mice subjected to TBI at 3.2 Gy and injected with hMSCs intravenously. Abbreviations: ALT: serum alanine aminotransferase; AST: serum aspartate aminotransferase. (a) Blood urea concentrations. ((b) and (c)) Blood AST and ALT concentrations, respectively. (d) Lipid peroxidation was assayed by measuring the amount of thiobarbituric acid (TBARS) formation. Irradiation induced oxidative stress (white histogram) and hMSC injection (gray histogram) decreased oxidative stress. All values are expressed as the mean and the standard error of the mean (SEM). The significance for all analyses was set at *P* < 0.01 (∗∗) and *P* < 0.001 (∗∗∗) and nonsignificance was noted as ns. Each group consisted of 5 animals (*n* = 5).

The BM-MSCs infusion in nonirradiated mice did not modify the blood urea and AST and ALT concentrations (7.84 ± 0.35 mmol/L; 72.67 ± 0.35 U/L and 17.20 ± 1.43 U/L, resp.) in comparison to the blood levels measured in nonirradiated and noninjected mice. No significant variation of these values was observed after hMSCs infusion in nonirradiated animals, suggesting the absence of toxicity of this graft. Biochemical analyses between irradiated noninjected animals and irradiated infused animals showed a decrease in blood AST and in ALT concentrations, 7 days after TBI (Figures 3(b) and 3(c)), while the blood urea concentration did not increase (Figure 3(a)). These observations suggested that systemic infusion of BM-MSCs restored preferentially the blood baseline levels of AST and ALT after TBI rather than the blood level of urea.

The BM-MSCs infusion in nonirradiated mice did not modify the blood urea and AST and ALT concentrations (7.84 ± 0.35 mmol/L; 72.67 ± 0.35 U/L and 17.20 ± 1.43 U/L, resp.) in comparison to the blood levels measured in nonirradiated and noninjected mice. No significant variation of these values was observed after hMSC infusion in nonirradiated animals, suggesting the absence of toxicity of this graft. Biochemical analyses between irradiated noninjected animals and irradiated infused animals showed a decrease in blood AST and in ALT concentrations, 7 days after TBI (Figures 3(b) and 3(c)), while blood urea concentrations did not increase (Figure 2(a)). These observations suggested that hMSCs restored preferentially the blood baseline levels of AST and ALT after irradiation rather than the blood level of urea.

### 3.4. hMSCs Decrease the Oxidative Stress Induced by Radiation

Oxidative stress in the cellular environment results in the formation of highly reactive and unstable lipid hydroperoxides. Decomposition of the unstable peroxides derived from polyunsaturated fatty acids results in the formation of malondialdehyde (MDA). Measuring MDA, we assayed lipid peroxidation in plasma in order to evaluate the modulation of oxidative stress after hMSC injection (Figure 3(d)). Irradiation-induced oxidative stress increased just after irradiation (days 2 and 3). hMSC injection decreased oxidative stress induced by irradiation significantly compared to irradiated mice noninjected mice (*P* < 0.001). These results imply that hMSCs have a protective effect against induced oxidative stress.

### 3.5. hMSC Engraftment in Liver

**Figure 4:**
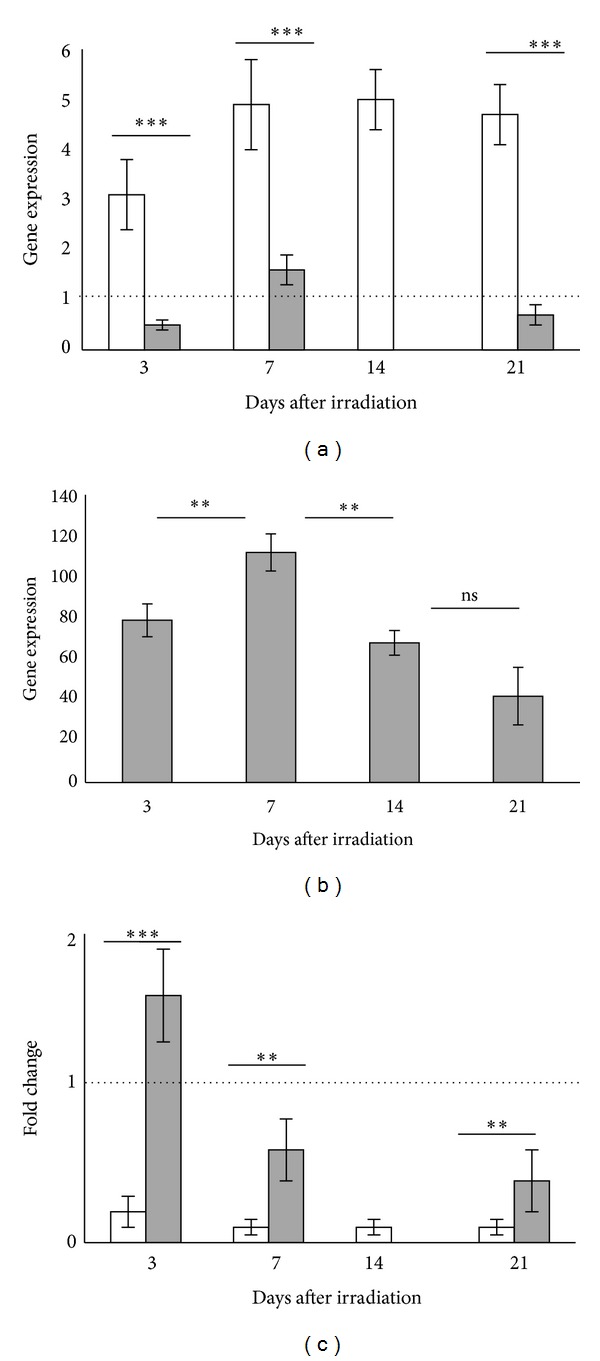
Expression of SDF1*α* and mir-27b in liver and CXCR4 in implanted hMSCs. (a) At 3 and 7 days after irradiation, the level of *SDF1* expression was significantly higher (*P* < 0.05) in liver engrafted with hMSCs (white histogram) compared to unimplanted liver (grey histogram). At 15 days, the comparison was not possible because all the livers tested contained hMSCs. (b) The level of *CXCR4* in hMSCs increased in vivo during the first days after injection and then decreased after 15 days. (c) The level of mir-27b in the liver of mice engrafted with hMSCs is less elevated than in liver ungrafted (Figure 4(c)). The level of mir-27b is more elevated in liver of mice ungrafted in comparison with liver containing hMSCs. The significance for all analyses was set at *P* < 0.01 (∗∗) and *P* < 0.001 (∗∗∗) and nonsignificance was noted as ns. Each group consisted of 5 animals (*n* = 5).

**Figure 5:**
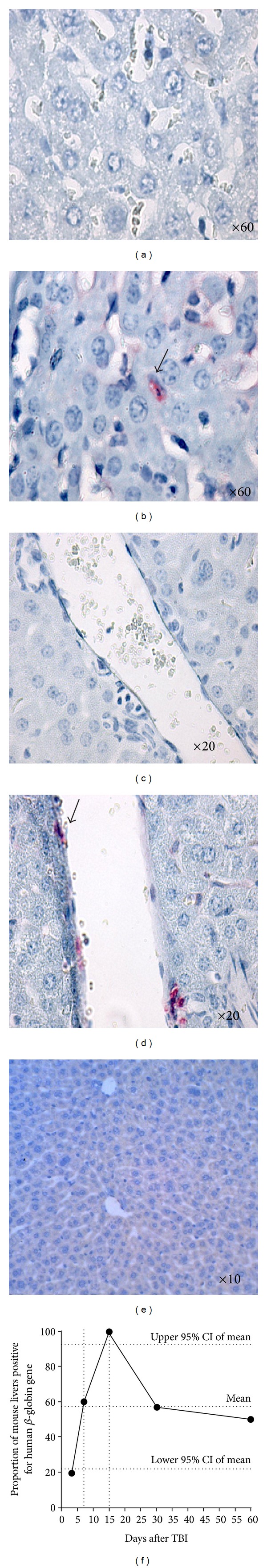
Human *β*-2-microglobulin immunostaining in mouse liver, 30 days after TBI. And percentage of mouse livers engrafted with hMSCs from 3 to 60 days after TBI. Immunostaining of hepatic cells (b), endothelium of portal vein (d) with human cells expressing beta-2-microglobulin stained in red, and respective negative controls ((a) and (c)). Isolated human cells can be seen between hepatic ((b) black arrow) and endothelial cells ((d) black arrow). (e) Negative control nonirradiated, noninjected with hMSCs (f).

Stromal cell-derived factor 1 (SDF1), which is secreted by cells within injured tissues, and its receptor C-X-C chemokine receptor type 4 (CXCR4) are necessary for the migration of MSCs to damaged tissues. MSCs in the bone marrow express high levels of *CXCR4*. However, *CXCR4* expression is markedly reduced during the ex vivo expansion of MSCs. In our experiment, 3 days after irradiation only 20% of the mice had hMSCs engrafted in liver the frequency increased to 50% at 7 days and reached 100% at 15 days (Figure 5(f)). In order to determine whether engraftment is related to the quantity of SDF1 secreted by liver; we compared the level of SDF1 expression in liver containing hMSCs to liver without engraftment of hMSCs (Figure 4(a)). At 3 and 7 days after irradiation, the level of *SDF1* expression was significantly higher (*P* < 0.05) in liver engrafted with hMSCs compared to unimplanted liver. At 15 days, the comparison was not possible because all the livers tested contained hMSCs. Results are expressed as fold increase expression as compared to unirradiated liver not injected with hMSCs.

Since MSCs lost CXCR4 during culture, we tested whether MSCs may increase *CXCR4 *expression in vivo, in contact with organ secreting SDF1 such as irradiated liver in our experiment (Figure 4(b)). Results are expressed as fold increase expression of hMSCs engrafted in liver as compared to hMSCs before injection and normalized to *GAPDH*. The level of *CXCR4* increased rapidly and significantly in vivo during the first days after injection and then decreased after 15 days (*P* < 0.05).

A recent report describes that mir-27b can suppress the directional migration of MSCs by downregulating SDF1*α* expression by binding directly to the SDF1*α* 3′UTR [44]. We compared the level of mir-27b in liver of mice engrafted with hMSCs to ungrafted liver (Figure 4(c)). The level of mir-27b was more elevated in the liver of ungrafted mice in comparison to that of the liver containing hMSCs (*P* < 0.05).

In our conditions, hMSCs might have acquired in vivo a high level CXCR4 expression and migrated in irradiated liver expressing high levels of *SDF1*α**. A high level of *SDF1*α** might be related to a low level of mir-27b. We conclude that hMSCs engraftment is related to a low level of mir-27b.

Figure 5(f) displays the proportion of mouse livers where hMSCs were detected using quantitative PCR of the human *β*-globin gene. The human *β*-globin gene signal was detected at each time point studied from 3 to 60 days after TBI. Human DNA was detected in 20% of livers at 3 days after TBI. The highest number of livers in which human DNA was detected was observed at 15 days after TBI. Three weeks after TBI, the proportion of liver in which human DNA was detected decreased. The number of livers positive, in PCR, for human gene presence appeared to stabilize at approximately 50% and the second month after irradiation. The colonization of the liver by hMSCs appeared to be maximal 15 days after radiation exposure. As shown in Figure 5(f), an exponential increase in the number of livers containing human DNA was detected during the period of reduction in the hepatic activity, the critical phase. This correlation suggests that there is a larger mobilization of the BM-MSCs towards the liver of irradiated mice during the critical phase of this tissue. Thus, the hMSCs' engraftment kinetics in the liver could be correlated to the reduction in hepatic activity.

To determine the quantity of human cells, quantitative PCR on the human *β*-globin gene was performed in livers from 3 to 60 days after TBI. The stippled lines represent the postirradiation period during which a reduction in the hepatic activity was observed (from 7 to 15 days after irradiation). From 20% (day 3) to 100% (day 15) of livers contain human DNA. A substantial engraftment of hMSCs in the mouse liver was measured during the period of reduction in the hepatic activity. hMSCs can have early beneficial effects on hepatic activity.

In a second step, we determined the frequency of hMSCs in the livers engrafted with hMSCs.  Table 3 lists the frequency of hMSCs in irradiated NOD/SCID mouse livers. The quantitative detection of human *β*-globin gene in mouse liver from 3 to 60 days after TBI showed a significant increase of hMSC engraftment during this period (Table 3). Between 15 and 30 days after TBI, the quantity of human cells in liver increased significantly by 28-fold (*P* = 0.001). Thus, it appears that the percentage of livers positive for human bone marrow-derived stromal cells is not correlated linearly with the quantity of human DNA detected.

Moreover, the hMSC infusion involved a return to the basic levels of urea and transaminases 7 days after TBI (Figure 2). Altogether, these results suggest that the hMSCs can act very precociously and in very small quantity. If hMSCs have a beneficial effect on the liver before the 15th day after irradiation, this suggests that it is not the quantity of hMSC engrafted that is important. The important aspect would be that these adult cells are present in small quantity. At 30 days after irradiation, the hMSC quantity detected remains high, which suggests a strong presence of hMSCs and therefore a possible proliferation of them in this organ.

### 3.6. Localization of hMSC Engrafted in the Liver by Immunohistology, 30 Days after TBI (Figures 5(a)–5(e))

To detect the hMSCs in situ, we used livers collected at 30 days after irradiation, when the highest quantity of human cells could be detected. To localize human cells in engrafted livers, we performed immunohistologic experiments using a human *β*-2-microglobulin specific antibody. Staining was carried out on livers collected in animals subjected to TBI, 30 days after exposure. Cells expressing the human *β*-2-microglobulin were observed either isolated between hepatic cells (Figure 5(b)) or in the endothelium of the portal vein (Figure 5(d)). Human cells were mainly localized in the perivascular region of the liver 30 days after TBI. In a previous study, we reported about the migration of hMSCs through the vascular wall and about an intravascular colonization under intima in the lungs [7]. These observations in the liver are in agreement with those published on the lungs and support the hMSCs' capacity to colonize the perivascular region of various tissues.

### 3.7. hMSC Differentiation in the Liver by RT-PCR

**Figure 6:**
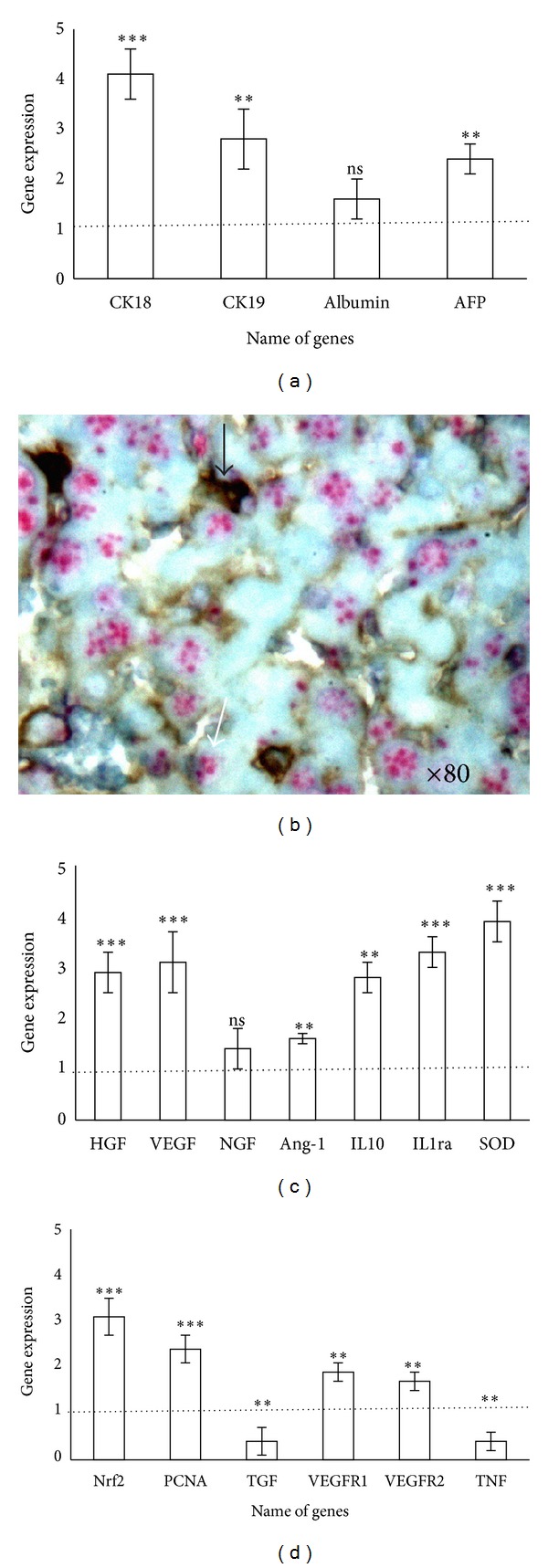
In situ hybridization analysis and gene expression of hMSCs implanted in liver. (a) To determine whether implanted hMSCs in vivo expressed liver specific genes hMSCs before injection was compared to hMSC gene expression 15 days after injection. RT-PCR values are representative of at least 5 replicates. Results are expressed as a fold increase in gene expression in hMSCs in liver compared to hMSCs before injection. Values greater than one indicate upregulation of the target genes and values less than one indicate their downregulation. (b) Determination of cell fusion. Black arrow indicates human cells beta-2-microglobulin stained. FISH analysis showing the mice transcription centers (white arrow). (Original magnification ×80.) (d) Determination of growth factors secreted by hMSCs implanted in mice liver. The significance for all analyses was set at *P* < 0.01 (∗∗) and *P* < 0.001 (∗∗∗) and nonsignificance was noted as ns. Each group consisted of 5 animals (*n* = 5).

At 15 days after transplant (when 100% of liver possessed human cell implantation) to study the differentiation of human cells, we measured human mRNA of mice liver engrafted with human cells. To control that primers amplified specifically human mRNA, we examined liver total RNA from 5 control mice which had not received hMSCs. Values greater than one indicate upregulation of the target genes and values less than one indicate their downregulation. To determine whether implanted hMSCs in vivo expressed liver specific genes (Figure 6(a)), we measured human mRNA of *CK18, CK19,* albumin, and *AFP*. Results are expressed as fold increase expression as compared to hMSCs before injection and normalized to GAPDH. The hMSC engraftment significantly increased (*P* < 0.05) the expression of *CK18* (4.1-fold), *CK19* (2.8-fold), and *AFP *(2.4-fold). The hMSCs expressed a hepatocyte phenotype.

In a second step, we controlled whether a cell fusion occurred between hMSCs and hepatocytes. We determined cell fusion. Human cells were stained with beta-2-microglobulin (black arrow) and a fluorescence in situ hybridization (FISH) analysis showed the mice transcription centers (white arrow). No fusion of human cells with mice hepatocytes was observed.

In a third step, we investigated whether hMSCs induced liver regeneration by secretion of growth factors or limitation of inflammation (Figure 6(c)). The data was expressed as fold increase in the mRNA level of hMSCs before injection compared to human cells after implantation in liver. HGF is important in the proliferation phase of hepatocyte. An increased of 2.3 fold of HGF was observed. *VEGF* increased 3.1-fold, *NGF* 1.4-fold, and Ang-1 1.6-fold. Anti-inflammatory gene expression *IL10* and *IL1ra* increased 2.8- and 3.3-fold, respectively (significantly, *P* < 0.05). The secretion of growth factors and the modulation of inflammation promote liver regeneration. The hMSC engraftment exerts a paracrine proliferative effect on endogenous hepatocytes and endothelial cells.

In a fourth step, by measuring gene expression of mice liver gene (Figures 6(c) and 6(d)), we controlled whether hMSCs decrease oxidative stress (*SOD, Nrf2*) and promote hepatocytes (PCNA, TGF, TNF) and endothelial proliferation (VEGF-R1, VEGF-R2). The expression of *SOD* (3.9-fold), *Nrf2* (3.1), *PCNA* (2.4), and *VEGFR1* (1.9), *VEGFR2* (1.7) increased and *TGF *(0.4) and *TNF* (0.4) decreased significantly (*P* < 0.05).

In conclusion, we found no fusion of human cells with mice hepatocytes but an increase of gene expression specific of hepatocyte cells was observed. The hMSC liver regeneration process operated by different mechanisms: (1) tissue differentiation, (2) secretion of growth factors, (3) angiogenesis, (4) downregulation inflammation, and (5) limitation of oxidative stress.

## 4. Discussion

**Figure 7:**
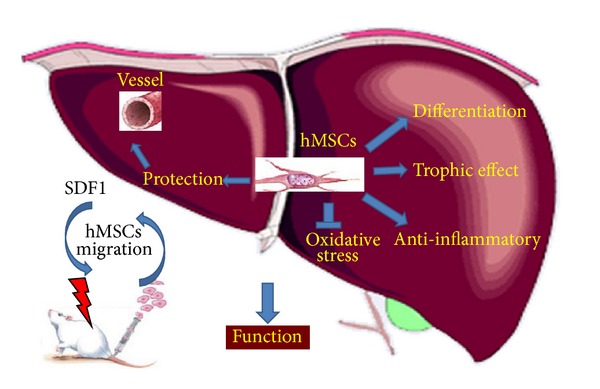
Effect of intravenous hMSCs injection on liver after total body irradiation (TBI). HMSCs migrate into liver, correcting radio-induced liver dysfunction by a simultaneous effect on hepatocyte, protection of vascular functions, differentiation, secretion of trophic, and anti-inflammatory factors.

Irradiation induced an increase of plasmatic transaminases levels, an increase of the apoptosis process in the liver vascular system, and an increase of oxidative stress. hMSC injection decreased transaminases levels, oxidative stress, and apoptotic cells and may reduce ROS production in the injured liver. HMSCs corrected radio-induced liver dysfunction by a simultaneous effect on hepatocyte differentiation, protection of vascular functions, secretion of anti-inflammatory, and trophic factors (Figure 7).

Seven days after TBI, the urea level was significantly lower in irradiated mice than in non irradiated mice or in mice irradiated and injected with hMSCs. In addition, the absence of variation in water and food intake of the animals suggests that the reduction in the urea level is related to hepatic insufficiency. The modification of the plasmatic concentrations of urea and transaminases correlating with hepatic activity, without variation of the renal activity, shows that the liver is more radiosensitive than the kidneys. The systemic hMSC infusion 24 hours after TBI appears to be beneficial to the hepatic activity. In mice subjected to TBI, hMSC infusion allowed for the maintenance of the basic plasmatic levels of urea, AST, and ALT. The beneficial effects of an intravenous (IV) MSC injection have also been observed in other experimental models. IV infusion of MSCs soon after acute myocardial infarction (MI) in swine was shown to improve left ventricular ejection fraction and to limit wall thickening in the remote noninfarcted myocardium, consistent with a beneficial effect on post-MI ventricular remodeling [45]. IV delivery of MSC prepared from adult BM reduces infarction size and ameliorates functional deficits of cerebral ischemia and spinal cord injury in rat models [46]. In a nonhuman primate model, the route of MSC delivery (intratissue or IV) does not modify the capacity of MSC engraftment [47].

SDF1 and CXCR4 are necessary for migration of MSCs to damaged tissues. *CXCR4* expression is reduced during the ex vivo expansion of MSCs. We have shown that the level of SDF1 expression was higher in liver engrafted with hMSCs compared to unimplanted liver. We tested whether MSCs may increase CXCR4 in vivo in contact with organ secreting SDF1 such as irradiated liver. In hMSCs, the level of CXCR4 increased rapidly and substantially in vivo during the first days after injection. Furthermore, the level of mir-27b is more elevated in the liver of ungrafted mice in comparison with the liver containing hMSCs. These results are in accordance with a recent report that describes that mir-27b can suppress the directional migration of MSC by downregulating *SDF1α* expression by binding indirectly to the *SDF1α* 3′UTR [44]. We have shown that hMSCs acquired in vivo a high level *CXCR4* expression and migrated in irradiated liver expressing a high level of *SDF1α*. A high level of *SDF1*α** might be related to a low level of mir-27b. We conclude that hMSC engraftment is related to a low level of mir-27b.

MSCs protect against injury by altering the oxidative microenvironment of the liver. *Nrf2* is a transcription factor that positively regulates the basal and inducible level of cytoprotective genes. *Nrf2* activation is protective against oxidative stress and induced SOD production which decreased ROS in liver [42]. We have shown that hMSC injection induced an increase in *Nrf2*, *SOD* gene expression which might reduce ROS production in the injured liver decreasing oxidative stress induced by irradiation. These results imply that hMSCs have a protective effect against induced oxidative stress.

To specifically identify human cells expressing *β*2-microglobulin, we determined that hMSCs preferentially localized in the endothelium of the portal vein and between hepatic cells. In accordance with a previous report [48], no fusion of human cells with mice hepatocytes and an increase of gene expression specific of hepatocyte were observed. Engrafted hMSCs expressed *CK18*, *CK19,* and *AFP* genes indicating a possible hepatocyte differentiation. The transdifferentiation ability of hMSCs into hepatocytes may play a role in the repair of injured liver. Nevertheless, these cells were limited to a small portion of total liver mass and were not sufficient to reverse the injury (a percentage of 2.5 is necessary for this process). Differentiation of hMSCs in hepatocytes participates in liver regeneration as previously reported [28].

The human cells injected intravenously were mainly localized in the perivascular region of the liver 30 days after TBI, as already reported in the lung [7]. The presence of hMSCs expressing *VEGF* in the perivascular region can confer a role of secreting cells to hMSCs in order to maintain the endothelial vascular cell functions. Furthermore, hMSCs upregulated gene expression of *VEGF-R1, R2* in liver. hMSCs promote neoangiogenesis after liver irradiation as previously reported after liver resection [49].

Recent work suggests that vascular endothelial function is significantly impaired due to oxidative stress mediated by the generation of oxygen-derived free radicals in response to chronic or acute inflammation [50]. Moreover, the endothelium is described as a central regulator of vascular and body homeostasis. The vascular endothelium is versatile and multifunctional. In addition to its role as a selective permeability barrier, it is involved in many synthetic and metabolic properties including the modulation of vascular tone and blood flow, the regulation of immune and inflammatory responses, and the regulation of coagulation, fibrinolysis, and thrombosis. Gaugler and colleagues reported that exposure to radiation can alter endothelial cells functions [51]. These radiation-induced negative effects could be playing a critical role in mediating organ dysfunction. Thus, hMSC localization to the perivascular region could protect the vascular endothelium by the secretion of cytokines and/or chemokines, which in turn stimulate or maintain the integrity of this fundamental structure by preventing the appearance of secondary negative effects in the liver. This protective mechanism could also apply to other tissues, as hypoxia has been shown to influence the localization of hMSCs [52].

The expression of growth factors by hMSCs in liver including *NGF*, *HGF*, and *Ang-1* and anti-inflammatory molecules (*L-10, IL-1ra*) related to an increasing cell proliferation in liver (increase of *PCNA* gene expression) might correct liver dysfunction and promote liver regeneration as previously published [29, 48]. NGF, HGF, and VEGF are reported to increase the intrinsic ability of hepatocytes to proliferate or to facilitate the breakdown of scar tissue [48]. TBI induced negative effect on the liver. hMSC systemic infusion significantly prevented the secondary hepatic effect, while the hMSC infusion itself did not induce noticeable toxic effects. This correlates with the report that MSC infusion can increase the survival of patients developing GVHD [23, 53]. These findings and clinical observations reinforce the assumption that MSCs are potent candidates for protecting against and repairing various damages [42, 44].

## Figures and Tables

**Table 1:** Primer references of studied genes.

Gene name	Reference
*GAPDH *	Mm99999915_g1
*CK18 *	Mm01601704_g1
*CK19 *	Mm00802090_m1
*Alb *	Mm00802090_m1
*AFP *	Mm00431715_m1
*Nrf2 *	Mm00477784_m1
*PCNA *	Mm00448100_g1
*TGFb1 *	Mm01178820_m1
*VEGFR1 *	Mm00438980_m1
*VEGFR2 *	Mm01222421_m1
*TNFa *	Mm00443260_g1

*miRNA U6 *	001973
*mir-27b *	000409

*GAPDH *	Hs00266705_g1
*HGF *	Hs01117422_g1
*SOD *	Hs00533490_m1
*VEGF *	Hs00900055_m1
*NGF *	Hs00171458_m1
*Ang-1 *	Hs01042023_g1
*IL10 *	Hs99999035_m1
*IL1-ra *	Hs00277299_m1

**Table 2:** FACS analysis of hMSCs at the second passage, before transplant from 10 bone marrows. Frequency of positive cells for specific markers of hMSCs: CD105 and CD73 and hematopoietic cell markers (CD45).

	BM mL	hMSCs (10^6^ cells)	% CD73	% CD105	% CD45
Mean	15.4	25.9	84.2	61.3	0.18
SEM	0.8	1.6	1.3	1.2	0.02

**Table 3:** Frequency of human cells in livers of irradiated (TBI) NOD/SCID mice after hMSC infusion. The frequency of hMSCs engrafted in liver was quantified using a PCR detection of the human *β*-globin gene in mouse liver from 3 to 60 days after TBI. The hMSC engraftment rate was significantly increased at 30 days in the liver following TBI, when compared to the 15th day. All values are expressed as the mean and the standard error of the mean (SEM), *n* = 10.

Days after TBI	3	7	15	30	60
% human cells (mean, *n* = 10)	0.07%	0.09%	0.11%	3.08%	2.52%
SEM	0.01	0.04	0.12	1.12	0.91

## References

[1] Pittenger MF, Mackay AM, Beck SC (1999). Multilineage potential of adult human mesenchymal stem cells. *Science*.

[2] Gao J, Dennis JE, Muzic RF, Lundberg M, Caplan AI (2001). The dynamic in vivo distribution of bone marrow-derived mesenchymal stem cells after infusion. *Cells Tissues Organs*.

[3] Devine SM, Cobbs C, Jennings M, Bartholomew A, Hoffman R (2003). Mesenchymal stem cells distribute to a wide range of tissues following systemic infusion into nonhuman primates. *Blood*.

[4] Chapel A, Bertho JM, Bensidhoum M (2003). Mesenchymal stem cells home to injured tissues when co-infused with hematopoietic cells to treat a radiation-induced multi-organ failure syndrome. *Journal of Gene Medicine*.

[5] Bensidhoum M, Chapel A, Francois S (2004). Homing of in vitro expanded Stro-1- or Stro-1+ human mesenchymal stem cells into the NOD/SCID mouse and their role in supporting human CD34 cell engraftment. *Blood*.

[6] Allers C, Sierralta WD, Neubauer S, Rivera F, Minguell JJ, Conget PA (2004). Dynamic of distribution of human bone marrow-derived mesenchymal stem cells after transplantation into adult unconditioned mice. *Transplantation*.

[7] François S, Bensidhoum M, Mouiseddine M (2006). Local irradiation not only induces homing of human mesenchymal stem cells at exposed sites but promotes their widespread engraftment to multiple organs: a study of their quantitative distribution after irradiation damage. *Stem Cells*.

[8] François S, Mouiseddine M, Mathieu N (2007). Human mesenchymal stem cells favour healing of the cutaneous radiation syndrome in a xenogenic transplant model. *Annals of Hematology*.

[9] Bey E, Prat M, Duhamel P (2010). Emerging therapy for improving wound repair of severe radiation burns using local bone marrow-derived stem cell administrations. *Wound Repair and Regeneration*.

[10] Horie M, Sekiya I, Muneta T (2009). Intra-articular injected synovial stem cells differentiate into meniscal cells directly and promote meniscal regeneration without mobilization to distant organs in rat massive meniscal defect. *Stem Cells*.

[11] Wu X, Huang L, Zhou Q (2005). Mesenchymal stem cells participating in ex vivo endothelium repair and its effect on vascular smooth muscle cells growth. *International Journal of Cardiology*.

[12] Coronel MF, Musolino PL, Villar MJ (2006). Selective migration and engraftment of bone marrow mesenchymal stem cells in rat lumbar dorsal root ganglia after sciatic nerve constriction. *Neuroscience Letters*.

[13] Natsu K, Ochi M, Mochizuki Y, Hachisuka H, Yanada S, Yasunaga Y (2004). Allogeneic bone marrow-derived mesenchymal stromal cells promote the regeneration of injured skeletal muscle without differentiation into myofibers. *Tissue Engineering*.

[14] Wang XY, Liu B, Yuan CH, Yao HY, Mao N (2003). Effect of bone marrow mesenchymal stem cells on hematopoietic differentiation of murine embryonic stem cells. *Zhongguo Shi Yan Xue Ye Xue Za Zhi*.

[15] Sato Y, Araki H, Kato J (2005). Human mesenchymal stem cells xenografted directly to rat liver are differentiated into human hepatocytes without fusion. *Blood*.

[16] Aurich I, Mueller LP, Aurich H (2007). Functional integration of hepatocytes derived from human mesenchymal stem cells into mouse livers. *Gut*.

[17] Ringe J, Kaps C, Burmester G, Sittinger M (2002). Stem cells for regenerative medicine: advances in the engineering of tissues and organs. *Naturwissenschaften*.

[18] Tuan RS, Boland G, Tuli R (2003). Adult mesenchymal stem cells and cell-based tissue engineering. *Arthritis Research and Therapy*.

[19] Pelled G, Turgeman G, Aslan H, Gazit Z, Gazit D (2002). Mesenchymal stem cells for bone gene therapy and tissue engineering. *Current Pharmaceutical Design*.

[20] Muguruma Y, Yahata T, Miyatake H (2006). Reconstitution of the functional human hematopoietic microenvironment derived from human mesenchymal stem cells in the murine bone marrow compartment. *Blood*.

[21] Koç ON, Gerson SL, Cooper BW (2000). Rapid hematopoietic recovery after coinfusion of autologous-blood stem cells and culture-expanded marrow mesenchymal stem cells in advanced breast cancer patients receiving high-dose chemotherapy. *Journal of Clinical Oncology*.

[22] Fouillard L, Bensidhoum M, Bories D (2003). Engraftment of allogeneic mesenchymal stem cells in the bone marrow of a patient with severe idiopathic aplastic anemia improves stroma. *Leukemia*.

[23] Le Blanc K, Ringdén O (2006). Mesenchymal stem cells: properties and role in clinical bone marrow transplantation. *Current Opinion in Immunology*.

[24] Ringdén O, Uzunel M, Rasmusson I (2006). Mesenchymal stem cells for treatment of therapy-resistant graft-versus-host disease. *Transplantation*.

[25] Caplan AI (1995). Osteogenesis imperfecta, rehabilitation medicine, fundamental research and mesenchymal stem cells. *Connective Tissue Research*.

[26] Horwitz EM, Prockop DJ, Fitzpatrick LA (1999). Transplantability and therapeutic effects of bone marrow-derived mesenchymal cells in children with osteogenesis imperfecta. *Nature Medicine*.

[27] Koç ON, Day J, Nieder M, Gerson SL, Lazarus HM, Krivit W (2002). Allogeneic mesenchymal stem cell infusion for treatment of metachromatic leukodystrophy (MLD) and Hurler syndrome (MPS-IH). *Bone Marrow Transplantation*.

[28] Ayatollahi M, Soleimani M, Tabei SZ, Salmani MK (2011). Hepatogenic differentiation of mesenchymal stem cells induced by insulin like growth factor-I. *World Journal of Stem Cells*.

[29] Zhao W, Li JJ, Cao DY (2012). Intravenous injection of mesenchymal stem cells is effective in treating liver fibrosis. *World Journal of Gastroenterology*.

[30] Fouraschen SM, Pan Q, de Ruiter PE (2012). Secreted factors of human liver-derived mesenchymal stem cells promote liver regeneration early after partial hepatectomy. *Stem Cells and Development*.

[31] Zhao L, Feng Z, Hu B, Chi X, Jiao S (2012). Ex vivo-expanded bone marrow mesenchymal stem cells facilitate recovery from chemically induced acute liver damage. *Hepatogastroenterology*.

[32] Mouiseddine M, François S, Souidi M, Chapel A (2012). Intravenous human mesenchymal stem cells transplantation in NOD/SCID mice preserve liver integrity of irradiation damage. *Methods in Molecular Biology*.

[33] Caplan AI (2013). Adult mesenchymal stem cells and the NO pathways. *Proceedings of the National Academy of Sciences of the United States of America*.

[34] Cho KA, Woo SY, Seoh JY, Han HS, Ryu KH (2012). Mesenchymal stem cells restore CCl_4_-induced liver injury by an antioxidative process. *Cell Biology International*.

[35] Muscaritoli M, Grieco G, Capria S, Iori AP, Fenelli FR (2002). Nutritional and metabolic support in patients undergoing bone marrow transplantation. *The American Journal of Clinical Nutrition*.

[36] Bourguignon MH (2000). The use of ionising radiations in medicine. A new era?. *Quarterly Journal of Nuclear Medicine*.

[37] Lorimore SA, Wright EG (2003). Radiation-induced genomic instability and bystander effects: related inflammatory-type responses to radiation-induced stress and injury? A review. *International Journal of Radiation Biology*.

[38] El-Sayed MH, El-Haddad A, Fahmy OA, Salama II, Mahmoud HK (2004). Liver disease is a major cause of mortality following allogeneic bone-marrow transplantation. *European Journal of Gastroenterology and Hepatology*.

[39] Dulley FL, Kanfer EJ, Appelbaum FR (1987). Venocclusive disease of the liver after chemoradiotherapy and autologous bone marrow transplantation. *Transplantation*.

[40] McDonald GB, Shulman HM, Wolford JL, Spencer GD (1987). Liver disease after human marrow transplantation. *Seminars in Liver Disease*.

[41] Ma SY, Au WY, Ng IO (2004). Hepatitic graft-versus-host disease after hematopoietic stem cell transplantation: clinicopathologic features and prognostic implication. *Transplantation*.

[42] Shi M, Zhang Z, Xu R (2012). Human mesenchymal stem cell transfusion is safe and improves liver function in acute-on-chronic liver failure patients. *Stem Cells Translational Medicine*.

[43] Kim SH, Kang KA, Zhang R (2008). Protective effect of esculetin against oxidative stress-induced cell damage via scavenging reactive oxygen species. *Acta Pharmacologica Sinica*.

[44] Lü MH, Li CZ, Hu CJ (2012). MicroRNA-27b suppresses mouse MSC migration to the liver by targeting SDF-1*α* in vitro. *Biochemical and Biophysical Research Communications*.

[45] Price MJ, Chou CC, Frantzen M (2006). Intravenous mesenchymal stem cell therapy early after reperfused acute myocardial infarction improves left ventricular function and alters electrophysiologic properties. *International Journal of Cardiology*.

[46] Nomura T, Honmou O, Harada K, Houkin K, Hamada H, Kocsis JD (2005). I.V. infusion of brain-derived neurotrophic factor gene-modified human mesenchymal stem cells protects against injury in a cerebral ischemia model in adult rat. *Neuroscience*.

[47] Mahmud N, Pang W, Cobbs C (2004). Studies of the route of administration and role of conditioning with radiation on unrelated allogeneic mismatched mesenchymal stem cell engraftment in a nonhuman primate model. *Experimental Hematology*.

[48] Li Q, Zhou X, Shi Y (2013). In vivo tracking and comparison of the therapeutic effects of MSCs and HSCs for liver injury. *PLoS ONE*.

[49] Nasir GA, Mohsin S, Khan M (2013). Mesenchymal stem cells and Interleukin-6 attenuate liver fibrosis in mice. *Journal of Translational Medicine*.

[50] Yamashita T, Shoge M, Oda E (2006). The free-radical scavenger, edaravone, augments NO release from vascular cells and platelets after laser-induced, acute endothelial injury in vivo. *Platelets*.

[51] Gaugler MH (2005). A unifying system: does the vascular endothelium have a role to play in multi-organ failure following radiation exposure?. *The British Journal of Radiology Supplement*.

[52] Rochefort GY, Vaudin P, Bonnet N (2005). Influence of hypoxia on the domiciliation of mesenchymal stem cells after infusion into rats: possibilities of targeting pulmonary artery remodeling via cells therapies?. *Respiratory Research*.

[53] Baron F, Storb R (2012). Mensenchymal stromal cells: a new tool against graft-versus-host disease?. *Biology of Blood and Marrow Transplantation*.

